# Temporal Alterations in White Matter in An *App* Knock-In Mouse Model of Alzheimer’s Disease

**DOI:** 10.1523/ENEURO.0496-23.2024

**Published:** 2024-02-22

**Authors:** Zachery D. Morrissey, Jin Gao, Aashutosh Shetti, Wenping Li, Liang Zhan, Weiguo Li, Igor Fortel, Takaomi Saido, Takashi Saito, Olusola Ajilore, Stephanie M. Cologna, Orly Lazarov, Alex D. Leow

**Affiliations:** ^1^Graduate Program in Neuroscience, University of Illinois Chicago, Chicago, Illinois 60612; ^2^Department of Psychiatry, University of Illinois Chicago, Chicago, Illinois 60612; ^3^Department of Anatomy & Cell Biology, University of Illinois Chicago, Chicago, Illinois 60612; ^4^Department of Electrical & Computer Engineering, University of Illinois Chicago, Chicago, Illinois 60607; ^5^Preclinical Imaging Core, University of Illinois Chicago, Chicago, Illinois 60612; ^6^Department of Chemistry, University of Illinois Chicago, Chicago, Illinois 60607; ^7^Department of Electrical & Computer Engineering, University of Pittsburgh, Pittsburgh, Pennsylvania 15261; ^8^Department of Bioengineering, University of Illinois Chicago, Chicago, Illinois 60607; ^9^Department of Radiology, Northwestern University, Chicago, Illinois 60611; ^10^Laboratory for Proteolytic Neuroscience, RIKEN Center for Brain Science, Wako 351-0198, Japan; ^11^Department of Neurocognitive Science, Institute of Brain Science, Nagoya City University, Nagoya 467-8601, Japan; ^12^Department of Computer Science, University of Illinois Chicago, Chicago, Illinois 60607

**Keywords:** white matter, *App* ^*N* *L* *-* *G* *-* *F* */* *N* *L* *-* *G* *-* *F*^, oligodendrocytes, myelin, Alzheimer's disease, DTI

## Abstract

Alzheimer’s disease (AD) is the most common form of dementia and results in neurodegeneration and cognitive impairment. White matter (WM) is affected in AD and has implications for neural circuitry and cognitive function. The trajectory of these changes across age, however, is still not well understood, especially at earlier stages in life. To address this, we used the *App^NL-G-F/NL-G-F^* knock-in (APPKI) mouse model that harbors a single copy knock-in of the human amyloid precursor protein (*APP*) gene with three familial AD mutations. We performed *in vivo* diffusion tensor imaging (DTI) to study how the structural properties of the brain change across age in the context of AD. In late age APPKI mice, we observed reduced fractional anisotropy (FA), a proxy of WM integrity, in multiple brain regions, including the hippocampus, anterior commissure (AC), neocortex, and hypothalamus. At the cellular level, we observed greater numbers of oligodendrocytes in middle age (prior to observations in DTI) in both the AC, a major interhemispheric WM tract, and the hippocampus, which is involved in memory and heavily affected in AD, prior to observations in DTI. Proteomics analysis of the hippocampus also revealed altered expression of oligodendrocyte-related proteins with age and in APPKI mice. Together, these results help to improve our understanding of the development of AD pathology with age, and imply that middle age may be an important temporal window for potential therapeutic intervention.

## Significance Statement

Alzheimer’s disease (AD) is a progressive neurodegenerative disorder that develops decades before onset of cognitive impairment. The trajectory of the pathology across age in white matter (WM), however, is still not well understood. Here, we used an *App^NL-G-F/NL-G-F^* knock-in mouse model to study WM using diffusion tensor imaging (DTI) and biochemical analyses. We observed reduced fractional anisotropy, a WM integrity proxy, in the hippocampus and anterior commissure between middle and late age. Notably, we observed changes in oligodendrocytes in middle age that preceded the changes in DTI. Together, this suggests that alterations in oligodendrocyte homeostasis may contribute to changes in WM in middle age. These results may serve as an important biomarker, therapeutic target, and provide new insight into the progression of AD.

## Introduction

Alzheimer’s disease (AD) is the most common form of dementia. The early mechanisms underlying the pathology of AD, however, are still not well understood. One of the major challenges is that alterations in the brain take place many years before a clear behavioral manifestation can be diagnosed. Thus, observations in mouse models of AD may provide important information on these early processes. White matter (WM)-related alterations have been implicated in AD (Bartzokis, 2011). One of the earliest descriptions of WM changes in AD was in *postmortem* histological experiments by Brun & Englund in the 1980s, where they observed topographical and symmetrical loss of myelinated axons and WM lesions in AD patients (Brun and Englund, 1986). The development of magnetic resonance imaging (MRI), and in particular diffusion tensor imaging (DTI) (Basser et al., 1994), has allowed for the non-invasive study of brain structure in detail. Before the onset of cognitive impairment, brain atrophy—particularly in the hippocampus—is arguably one of the most reliable markers for AD (Fox et al., 1996; Ridha et al., 2006; Webster et al., 2014). DTI studies have also provided evidence that, in addition to changes in gray matter (GM) (Weston et al., 2015), WM is also degenerated in AD (Bozzali, 2002; Lo Buono et al., 2020). Furthermore, evidence suggests that alterations in WM appear early in the course of the disease, even before the well-characterized atrophy and neurodegeneration in GM (Fischer et al., 2015; Nasrabady et al., 2018). Thus, understanding the trajectory of WM changes may help us to better understand the development of AD and identify potential biomarkers for therapeutic intervention.

During development, there are orchestrated waves of oligodendrogenesis and myelination (El Waly et al., 2014). Originally thought to be static after development, oligodendrogenesis and myelination continue throughout adulthood (Blakemore, 1972; Kaplan and Hinds, 1980; Menn et al., 2006; El Waly et al., 2014), and is homeostatically regulated (Hughes et al., 2013; Hill et al., 2018). Recently, it has been found that adult oligodendrogenesis and myelination are actively involved in learning and memory (McKenzie et al., 2014; Steadman et al., 2020; Chen et al., 2021); thus, experience-dependent oligodendrocyte and myelin plasticity are likely important for healthy cognitive function (Williamson and Lyons, 2018; Xin and Chan, 2020; McNamara and Miron, 2021). Therefore, disruptions in WM and oligodendrocyte homeostasis could have an important role in cognitive decline in AD.

A small percentage of AD patients have mutations in the amyloid precursor protein (*APP*) and presenilin-1 (*PSEN1*) and presenilin-2 (*PSEN2*) genes that result in an autosomal dominant form of the disease, known as familial AD (FAD). Despite the low prevalence of FAD, study of the genetics of these FAD patients has led to the development of many commonly used AD mouse models (Webster et al., 2014). Here, we used the *App^NL-G-F/NL-G-F^* knock-in (APPKI) mouse model of AD developed by Saito et al. (2014) that contains a single copy knock-in of human *APP* with the Swedish (K670N, M671L), Arctic (E693G), and Beyreuther/Iberian (I716F) FAD mutations. We performed *in vivo* DTI of APPKI mice in early (4 months), middle (10 months), and late (>15 months) age groups to measure the macroscale structural properties of the brain across age from just before the onset of Aβ plaque deposition and cognitive impairment. We observed that APPKI mice had reduced fractional anisotropy (FA) in multiple regions between middle and late age, suggesting that this time window is when potential microstructural impairment occurs. In 10-month-old mice, we observed that APPKI mice had a greater number of oligodendrocytes in the hippocampus and anterior commissure (AC). Despite this, we observed a reduction in lipid staining in the AC, suggesting that more oligodendrocytes did not correspond to more myelin. Furthermore, proteomics analysis of the hippocampus showed multiple oligodendrocyte-related proteins with altered expression by age and in APPKI. Our results suggest that there are WM-related alterations in APPKI mice across age, and that changes in the oligodendrocyte population precede observed changes in DTI. Together, these results offer further evidence for the contribution of myelin and oligodendrocyte dynamics to the pathological development of AD.

## Materials and Methods

### Animals

Animal protocols were approved by the University of Illinois Chicago Institutional Animal Care and Use Committee. Mice used in this study were from a C57Bl /6 background. APPKI mice were generated by Saito et al. (2014). Tail samples were obtained from mice at time of weaning to test for APPKI genotype. Animals were housed in standard housing conditions with up to five mice per cage on a 12 h light–dark cycle and allowed to feed and drink *ad libitum*. Details for mice scanned in this study are shown in Extended Data Table 1-1.

### *In vivo* MRI acquisition

Animals were anesthetized using 1–2% isoflurane and imaged using a 9.4 T Agilent MRI system (Santa Clara, California, USA) as described previously (Morrissey et al., 2023). Briefly, mice were secured using a bite bar to restrict head motion, and ambient temperature and respiratory rate were monitored continuously during the scan using an SAII gating and monitoring system for small animals (SA Instruments, New York, USA). A *T*_2_ − weighted fast-spin echo sequence was acquired using the following parameters: TR = 2,000 ms, TE = 10 ms, echo train length = 8, slice thickness =1 mm, number of slices = 20, FOV = 19.2 mm × 19.2 mm, matrix size =128 × 128, acquisition time = 2 min 12 s.

A 3D shimming was performed prior to diffusion-weighted imaging (DWI) to mitigate magnetic field inhomogeneity, with both first- and second-order shims optimized in a user-defined shim voxel (20 mm × 20 mm × 20 mm) covering the brain volume for DWI (Vanzijl et al., 1994). DWI images were obtained using a 12-direction gradient table and *b*_0_ images (TR = 1,800 ms, TE = 23.22 ms, slice thickness =1 mm, number of slices =10, number of averages = 32, *b* = 1,000 s/mm^2^, FOV = 19.2 mm × 19.2 mm, matrix size =128 × 128, acquisition time =1 h 42 min 35 s).

### DTI analysis

**Figure 1.**
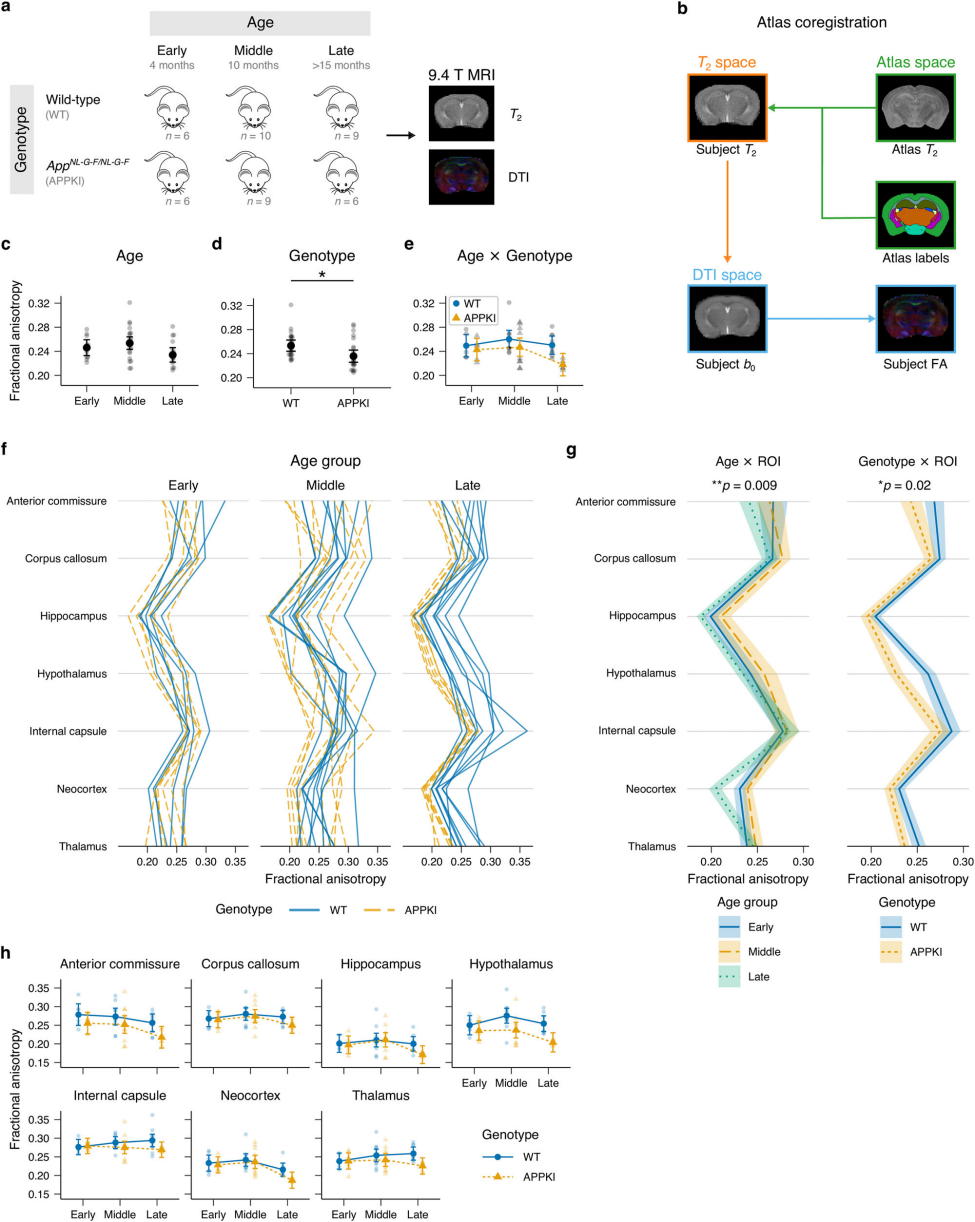
MRI experiment design and *in vivo* FA measurements by age, genotype, and region of interest (ROI). ***a***, *In vivo* MRI scanning paradigm. Wild-type and APPKI female mice were scanned using a 9.4 T MRI system for *T*_2_-weighted and 12-direction DTI sequences. Mice were scanned in early (4 months), middle (10 months), and late (>15 months) age groups. Descriptive statistics for mice is shown in Extended Data Table 1-1. ***b***, Atlas coregistration scheme. High resolution *T*_2_-weighted images from the atlas were registered to each subject’s *T*_2_-weighted image. Coregistration was performed to align each subject’s *T*_2_-weighted image to their corresponding *b*_0_ image from the DWI sequence. The atlas labels were then registered to each subject’s subject space by concatenating the previous two transformation matrices. The mean FA was used as the subject measure for each ROI. ***c***–***e***, Main effects of age (***c***) and genotype (***d***) and age × genotype interaction (***e***) for three-way mixed ANOVA model. Gray points represent mean FA for subject. Black points (***c*** and ***d***) and colored points (***e***) represent estimated marginal means for each group. Error bars represent 95% CI. ***f***, Mean FA measurements for each subject for each region. Lines connect observations from each subject. ***h***, Age × ROI and genotype × ROI interactions for the three-way mixed ANOVA model. Lines connect mean FA measurements for each group. Shaded area represents Cousineau–Morey-corrected 95% CI. ***h***, Mean FA measurements for each genotype across age by ROI. Colored points represent estimated marginal means for each group. Gray points represent individual subject means. Error bars represent 95% CI. Additional statistics are provided in Extended Data Table 1-(2-7).

*T*_2_ − weighted and DWI images were converted from raw digital imaging and communications in medicine format to Neuroimaging Informatics Technology Initiative (NIfTI) format using dcm2niix (Li, 2016). A brain mask was then created for both *T*_2_ − weighted and DWI images by manually outlining the brain using fsleyes (version 0.26.4) from the FMRIB Software Library (FSL) 6.0 (Smith et al., 2004). The diffusion tensor model was applied using the dtifit program from FSL (Jenkinson et al., 2012). The Waxholm space atlas (Johnson et al., 2010) was used to register atlas labels to each subject space by doing a two-step registration using the FSL flirt linear registration tool with 12 degrees of freedom (Fig. 1*b*). First, the high resolution *T*_2_ − weighted atlas image was registered to each subject’s *T*_2_ − weighted image. Next, the subject’s *T*_2_ − weighted image was registered to their respective *b*_0_ DWI image. Finally, the transformation matrices from these two steps were concatenated to transform the atlas labels to the subject’s *b*_0_ image using nearest neighbor interpolation. The FA output from dtifit was used with the registered atlas labels to measure the mean FA values for each region for each subject.

### Immunohistochemistry

Mice were anesthetized with isoflurane and transcardially perfused with ice-cold phosphate-buffered saline (PBS) followed by 4% w/v paraformaldehyde (PFA) to fix the brain. The brain was then extracted and placed in 4% w/v PFA to fix overnight at 4°C. The following day, brains were transferred to 30% w/v sucrose in PBS at 4°C for at least 3 d until the brain was fully submerged. Brains were frozen and coronal sections were cut at 50 μm thickness using a Leica SM2010R microtome. Free-floating sections were stored in 96-well plates containing a cryoprotectant composed of 30% v/v ethylene glycol and 30% v/v glycerol in PBS at −20°C.

Free-floating sections were washed three times for 5 min with PBS and mounted onto glass slides. Heat-mediated antigen retrieval was performed using 10 mM sodium citrate (pH 6) with 0.05% v/v Tween-20 at 95°C for 10 min. Slides were cooled to room temperature for 20 min and washed with PBS. Permeabilization and blocking were performed by adding PBS with 0.3 M glycine, 0.25% v/v Triton X-100, 5% v/v normal donkey serum, and 2% w/v bovine serum albumin for 1 h. Primary antibodies were then added in blocking buffer solutions at the specified dilutions and incubated for 3 d at 4°C. Primary antibodies used: 1:100 rabbit anti-OLIG2 (abcam ab109186) and 1:200 goat anti-platelet-derived growth factor receptor α (PDGFRA; R&D Systems AF1062). The sections were washed three times for 5 min with PBS. The secondary antibodies were added to blocking buffer at the specified dilutions and incubated for 1 h at room temperature under darkness. Secondary antibodies: 1:500 donkey anti-rabbit Cy3 (Jackson Immuno 711–165–152) and 1:500 donkey anti-goat Alexa Fluor 488 (Jackson Immuno 705–545–147). Sections were washed three times for 5 min with PBS; during the second wash, 5 μg/mL DAPI was added to stain nuclei. Sections were washed with 70% ethanol for 5 min and autofluorescence eliminator reagent (Millipore 2160) was added drop-wise to each section. Sections were then washed with 70% ethanol twice for 2 min. After the last wash, sections were allowed to dry and glass coverslips were mounted with polyvinyl alcohol DABCO (PVA-DABCO) antifade. The coverslips were sealed with clear nail polish, and slides were stored in a slide box at 4°C until imaged.

For Fluoromyelin Red staining, free-floating 50 μm coronal sections were washed three times for 5 min with PBS. Sections were permeabilized with PBS containing 0.3 M glycine and 0.1% v/v Triton X-100 for 20 min. Sections were incubated with 1:300 Fluoromyelin Red (ThermoFisher F34652) for 3 h. Sections were washed three times with PBS for 10 min. During the second wash, 5 μg/mL DAPI was added to stain nuclei. Sections were mounted onto slides and glass coverslips were mounted using PVA-DABCO and sealed as described above.

### Stereological quantification

Wild-type and APPKI serial 50 μm coronal sections were selected for stereological quantification of oligodendrocytes and oligodendrocyte precursor cells (OPCs) using the markers OLIG2 and PDGFRA using a Zeiss Axio Imager microscope system. The experimenter was blinded to the animal genotype during counting. The optical fractionator workflow from Stereo Investigator version 2020.2.3 (MicroBrightfield, Inc.) was used for quantification (Stereo Investigator, 2020). The ROIs were demarcated at 5 × magnification and quantified at 63 × magnification. The estimated number of cells for each marker was normalized to the ROI volume for each subject.

The parameters used for the optical fractionator protocol in Stereo Investigator are shown for the AC in Extended Data Table 2-1 and for the hippocampus in Extended Data Table 3-1. For each animal, the estimated number of cells *N* for each marker is defined as

(1) $$N=\sum_{i=1}^{n}Q_{i}^{-}\cdot \frac{\bar{t}}{h}\cdot \frac{1}{asf}\cdot \frac{1}{ssf}$$

for *n* sections, where $Q_{i}^{-}$ is the raw number of cells counted for the *i*th section, $\bar{t}$ is the mean section thickness, *h* is the optical disector height, *asf* is the area sampling fraction, and *ssf* is the section sampling fraction (Gundersen and Jensen, 1987; Gundersen et al., 1999; Keuker et al., 2001; Stereo Investigator, 2020). The estimated number of cells was normalized to the estimated volume of each region for each animal. Gundersen’s coefficient of error (CE) is defined as

(2) $$\mathrm{CE}=\frac{\sqrt{\;{\mathrm{Var}}_{\mathrm{total}}}}{s^{2}},$$

where $s^{2}=\sum_{i=1}^{n}Q_{i}^{-}$ is the variance due to noise for *n* sections, and Var_total_ = *s*^2^ + Var_SRS_ is the total variance, where Var_SRS_ is the systematic random sampling variance, defined as

(3) $${\mathrm{Var}}_{\mathrm{SRS}}=\frac{3\left(\left(\sum_{i=1}^{n}{\left(Q_{i}^{-}\right)}^{2}\right)-s^{2}\right)-4\sum_{i}^{n-1}Q_{i}^{-}Q_{i+1}^{-}+\sum_{i=1}^{n-2}Q_{i}^{-}Q_{i+2}^{-}}{240}.$$

### Western blot analysis

Mice were anesthetized with isoflurane and transcardially perfused with ice-cold PBS. The brain was extracted and placed into a Petri dish on ice containing ice-cold PBS. The hippocampus was dissected and snap-frozen in microcentrifuge tubes in liquid nitrogen and stored at −80°C until processed. Hippocampus tissue was thawed on ice, homogenized, and lysed in radioimmunoprecipitation assay buffer with protease and phosphatase inhibitors (Sigma P8340–5ML, Thermo 78420). Cells were lysed by sonicating three times for 15 s at 30% amplitude. Lysates were centrifuged at 16,000*g* for 15 min at 4°C and the supernatant was transferred to sterile microcentrifuge tubes. Protein estimation of lysate samples was performed using a bicinchoninic acid (BCA) assay (Thermo 23223 and 23224). Samples were prepared for sodium dodecyl sulphate (SDS)-PAGE at 1 μg/μL concentration with 10 × reducing agent (Invitrogen B0009) and 4 × sample buffer (Invitrogen B0007).

Samples were boiled at 95°C for 5 min prior to SDS-PAGE. SDS-PAGE was performed using 4–12% Bis–Tris polyacrylamide gels (Invitrogen NW04122BOX) at 200 V for 22 min. An iBlot2 dry blotting system (Thermo IB21001) was used to transfer samples from the polyacrylamide gel to a nitrocellulose membrane (20 V for 1 min, 23 V for 4 min, and 25 V for 2 min). Membranes were blocked with 5% w/v non-fat dairy milk powder in Tris-buffered saline with 0.1% v/v Tween-20 (TBST) for 1 h and incubated with primary antibodies at the specified dilutions in blocking buffer overnight at 4°C. Primary antibodies: 1:1,000 rabbit anti-OLIG2 (abcam ab109186) and 1:5,000 mouse anti-actin (Invitrogen MA5-11869). Membranes were washed three times for 15 min with TBST and incubated with horseradish peroxidase (HRP)-conjugated secondary antibodies at the specified dilutions for 1 h at room temperature. Secondary antibodies used: 1:10,000 anti-mouse IgG HRP (Promega W402B) and 1:10,000 anti-rabbit IgG HRP (Promega W4018). Membranes were then washed three times for 15 min with TBST. SuperSignal West PICO Plus chemiluminescent substrate (Thermo 1863094 and 1863095) was applied to the membranes and the membranes were exposed onto autoradiography films in a dark room.

Autoradiography films were scanned to a computer and the images were analyzed using FIJI (Schindelin et al., 2012). Images were converted to grayscale and the Analyze > Gels tool was used to estimate the gray value density for each band. The gray value density for the protein of interest was then calculated relative to the actin loading control for each sample. The relative values were then normalized to the mean of the wild-type group for comparing across groups.

### Microscopy

Confocal images were acquired using a Zeiss LSM 710 confocal microscope. Maximum intensity *z*-projections were used for representative images. Postprocessing of representative images was performed using the brightness/contrast, remove outliers, and subtract background tools in FIJI (Schindelin et al., 2012). The 4 × tile-stitched images of Fluoromyelin Red staining were acquired using a Keyence BZ-X800 microscope. For Fluoromyelin Red fluorescence intensity analysis, *z*-stack images of midline AC were acquired using the same imaging parameters, and sum intensity *z*-projections were used for quantification.

### Mouse hippocampal lysis and protein digestion for proteomics

Three male wild-type C57Bl/6 and three male *App^NL-G-F/NL-G-F^* mice were used for proteomics analysis. The mouse hippocampus was lysed in 5% SDS and 50 mM triethylammonium bicarbonate (TEAB) supplemented with SIGMAFAST^TM^ protease inhibitor tablets (S8820, Sigma). The protein concentration was determined by BCA (ThermoFisher) using a 96-well plate and analyzed on the VERSA max tunable microplate reader. To create an unbiased pool for normalization across multiple tandem mass tag (TMT) kits, an equal volume of lysates from one-year-old mouse hippocampal samples were combined (Romero et al., 2010). A total of 100 μg of proteins were first reduced with 20 mM dithiothreitol at 95°C for 10 min and then alkylated with 40 mM of iodoacetamide at room temperature in the dark for 30 min. The proteins were subsequently acidified with phosphoric acid and loaded onto the S-trap Micro Spin Column (Protifi, Huntington, New York). Following multiple washes with the S-trap binding buffer (90% methanol and 100 mM of TEAB), trypsin in 50 mM TEAB was introduced into the column and incubated at 37°C overnight. The digested peptides were eluted using a sequence of solutions: 50 mM TEAB, 0.1% formic acid (FA), and 50% acetonitrile (ACN) with 0.1% FA. The peptides were dried down for subsequent TMT isobaric tag labeling.

### TMT labeling and liquid chromatography-MS analysis

The peptides were reconstituted into 100 μL of 100 mM TEAB, and each TMT label reagent was resuspended into 41 μL of ACN. The labeling of peptides from each sample was carried out in accordance with the manufacturer’s instructions, as detailed in Extended Data Table 3-2. Following labeling, the samples were combined into a single microcentrifuge tube, dried *in vacuo*, and fractionated into 60 fractions using high pH reversed-phase liquid chromatography (LC). To reduce the complexity, these fractions were concatenated into 20 fractions by combining 3 fractions for every 20, and once again dried *in vacuo* (Huang et al., 2020). Each fraction was resuspended into 0.1% v/v FA and injected into a Thermo NanoViper trap column (75 μm × 20 mm, 3 μm C18, 100 Å) (Thermo Fisher Scientific) installed on an Agilent 1260 Infinity nanoLC system (Agilent Technologies) and processed as described previously (Golding et al., 2023). All raw mass spectrometry data can be accessed on the MassIVE data repository (http://massive.ucsd.edu) with project ID MSV000092969.

### Protein identification

Raw files from the LC-MS analysis were imported into Proteome Discoverer 2.3 (Thermo Fisher Scientific). These files were processed using the Sequest HT search engine and matched against the SwissProt *Mus musculus* database, which was downloaded in December 2019. In the search parameters, trypsin was specified as the protease, allowing for up to two missed cleavages, and sequences with lengths between 6 and 144 amino acids were considered. The mass error tolerances for precursor and fragment masses were set at 10 ppm and 0.02 Da, respectively. Masses with charges of 1 and larger than 6 were excluded from MS/MS analysis. Dynamic modifications taken into account during the analysis included oxidation (+15.995 Da; M), TMT (+229.163 Da; S, T), and acetylation (+42.011 Da; N-terminus). Static modifications included TMT6plex (+229.163 Da; any N-terminus) and carbamidomethylation (+57.021 Da, C). The quantification of identified proteins was carried out using the MSstatsTMT package, developed by Huang et al. (2020) within the R programming language. All samples were normalized to the unbiased pool sample. Detailed protein identifications are provided in Extended Data Tables 3-4 and 3-5.

### Protein annotation and abundance analysis

UniProt accession numbers for each peptide were annotated using the Org.Mm.eg.db and musculus_gene_ensembl databases from the biomaRt package using the R programming language. Non-specific peptides were removed. Principal component analysis (PCA) was performed on the scaled abundance values for each protein using the prcompfunction in R. The CellMarker (CM) 2.0 database (Hu et al., 2023) was used to identify oligodendrocyte-specific proteins using the query “mouse” + “brain” + “oligodendrocyte”. Heatmaps for CM protein lists were made using the ComplexHeatmap package in R to compare the scaled abundance values across all samples for each protein. The “CM score” in heatmaps was provided by the CM database “count” field in search results for each gene. Slopegraphs were made by calculating the mean scaled abundance values within each group and calculating the difference between APPKI and wild-type within each age group.

### Experimental design and statistical analysis

For FA analyses, a three-way mixed Type II ANOVA was performed using genotype and age group as between-subject variables and the brain ROI as a within-subject variable. The main effects of age, genotype, and ROI, as well as all interaction effects were performed accounting for the within-subject error by ROI. Violations of sphericity were corrected for using Greenhouse-Geisser correction. Statistical analyses were performed using the R programming language version 3.6.3 (R Core Team, 2018). Mixed ANOVA analyses were performed using the afex package (Singmann et al., 2021). *Post hoc* pairwise comparisons were performed using the emmeans package (Lenth, 2021). For Western blot and stereology quantification, Student’s independent *t*-test was used to compare groups. Fluoromyelin Red fluorescence intensity quantification was performed by pooling 40 × sum intensity *z*-projection image pixel values from each subject within group. The Kolmogorov–Smirnov (KS) test was used for testing the difference in distributions between groups.

For proteomics analyses, a linear mixed model (LMM) was used to test for the fixed effects of age and genotype across oligodendrocyte-related proteins using the lme4 (Bates et al., 2015) and lmerTest (Kuznetsova et al., 2017) packages in R. The abundance of each protein was standardized. The abundance *y*_*ij*_ of the *j*th oligodendrocyte-related protein for the *i*th subject was modeled allowing for a random slope and intercept for age and genotype for each protein, that is,

(4) $$\begin{array}{rl} y_{ij} & =(\beta_{0}+u_{0j})+\left(\beta_{1j}+u_{1j}\right){\mathrm{Age}}_{ij}+\left(\beta_{2j}+u_{2j}\right){\mathrm{Genotype}}_{ij}\\ & \;+\left(\beta_{3j}+u_{3j}\right){\left(\mathrm{Age}\times \mathrm{Genotype}\right)}_{ij}+\varepsilon_{ij} \end{array}$$

for subjects *i* = 1, …, *N*, and proteins *j* = 1, …, *P*, where age and genotype are treated as fixed effects. This was modeled in R as zscore ∼ Age * Genotype + (1 + (Age * Genotype) | Symbol). *Post hoc* pairwise comparisons were performed with the emmeanspackage (Lenth, 2021) with Tukey’s adjustment for multiple comparisons using emmeans(., list(pairwise ∼ Age * Genotype), adjust = “tukey”)).

Comparisons of protein abundance between groups were performed using Student’s independent *t*-test. Benjamini–Hochberg’s false discovery rate was used to correct for multiple comparisons for each comparison.

### Software

Neuroimaging analysis and fluorescence quantification were performed using FSL (Jenkinson et al., 2012) and Python version 3.8 (Van Rossum and Drake Jr, 1995) from the Anaconda distribution (Anaconda, 2018) and associated scientific computing libraries, including numpy (Harris et al., 2020), scipy (Virtanen et al., 2020), pandas (McKinney, 2010), scikit-learn (Pedregosa et al., 2011), nibabel (Brett et al., 2020), and nipype (Gorgolewski et al., 2011). Visualization was performed using Python with matplotlib (Hunter, 2007) and seaborn (Waskom, 2021), and R with ggplot2 (Wickham, 2016) and afex (Singmann et al., 2021) packages. Arrangement of figures was done using GNU Image Manipulation Program version 2.8.16 (Kimball et al., 2016) and Inkscape version 0.92 (Inkscape, 2017). Liquid_Chromatograph_Mass_Spectrometer icon provided by https://bioicons.com/ by DBCLS https://togotv.dbcls.jp/en/pics.html is licensed under CC-BY 4.0 Unported https://creativecommons.org/licenses/by/4.0/.

## Results

### Reduced FA in APPKI mice in late age

We performed *in vivo* DTI of female wild-type and APPKI mice in early (4 months), middle (10 months), and late (>15 months) age groups (Extended Data Table 1-1) in order to study changes in brain structure with age in the context of FAD. After fitting the tensor model to each image, we measured the mean FA in each brain ROI as a proxy of microstructural integrity (Fig. 1). A three-way mixed Type II ANOVA was performed to test for differences in FA using age group (early, middle, and late) and genotype (wild-type, APPKI) as between-subject factors and treating each ROI as a repeated measure (Fig. 1, Extended Data Table 1-2). There was a statistically significant interaction between age and ROI (*F*(7.28, 145.55) = 2.76, $\eta_{g}^{2}=0.026$, *p* = 0.009; Extended Data Table 1-3), as well as a statistically significant interaction between genotype and ROI (*F*(3.64, 145.55) = 3.14, $\eta_{g}^{2}=0.015$, *p* = 0.02; Fig. 1*g*, Extended Data Tables 1-2 and 1-4; additional statistics provided in Extended Data Tables 1-5–1-7). Overall, mice in the late age group and APPKI mice had lower FA compared to other groups. In particular, we observed the largest reductions in FA in the AC, hippocampus, hypothalamus, and neocortex, suggesting that there are microstructural changes in these regions between middle and late age.

10.1523/ENEURO.0365-23.2023.t1-1 Table 1-1 Descriptive statistics of mouse subjects scanned using MRI. The number of mice (*N*) and mean and standard deviation (SD) age in months is displayed. For each age group, a Mann-Whitney non-parametric test was performed to test if there were statistically significant differences in age between genotypes. Download Table 1-1, XLSX file.

10.1523/ENEURO.0365-23.2023.t1-2 Table 1-2 Three-way (Type II) mixed ANOVA model. Age group (early, middle, late) and genotype (wild-type, APPKI) were modeled as between-subject factors. Brain ROIs were modeled as a within-subject factor for each subject. Values are shown for the degrees of freedom (df), mean squared error (MSE), *F*-statistic, effect size: generalized *η*^2^ (GES; $\eta_{g}^{2}$), and *p*-values. The degrees of freedom and *p*-values were corrected for violations of sphericity using Greenhouse-Geisser (GG) correction. Significance (sig.) symbols: ***p < 0.001; **p < 0.01; *p < 0.05; + *p* < 0.1. Download Table 1-2, XLSX file.

10.1523/ENEURO.0365-23.2023.t1-3 Table 1-3 Age × ROI interaction follow-up comparisons by region. Tukey’s *post hoc* test was used to correct for multiple comparisons. Significance (sig.) symbols: ***p < 0.001; **p < 0.01; *p < 0.05; + *p* < 0.1. Download Table 1-3, XLSX file.

10.1523/ENEURO.0365-23.2023.t1-4 Table 1-4 Genotype × ROI interaction. Tukey’s *post hoc* test was used to correct for multiple comparisons. Significance (sig.) symbols: ***p *<* 0.001; **p < 0.01; *p < 0.05; + *p <* 0.1. Download Table 1-4, XLSX file.

10.1523/ENEURO.0365-23.2023.t1-5 Table 1-5 Age × ROI pairwise comparison. Benjamini-Hochberg’s method was performed to correct for multiple comparisons. Significance (sig.) symbols: ***p < 0.001; ** *p* < 0.01; *p < 0.05; + *p* < 0.1. Download Table 1-5, XLSX file.

10.1523/ENEURO.0365-23.2023.t1-6 Table 1-6 Planned contrasts for Age × Genotype interaction. Benj amini-Hochberg’s method was used to correct for multiple comparisons. Significance (sig.) symbols: *p < 0.05. Download Table 1-6, XLSX file.

10.1523/ENEURO.0365-23.2023.t1-7 Table 1-7 (Age × Genotype) | ROI interaction. Significance (sig.) symbols: **p < 0.01; + *p* < 0.1. Download Table 1-7, XLSX file.

### APPKI mice show greater oligodendrocytes, less myelin intensity, in the AC in middle age

**Figure 2.**
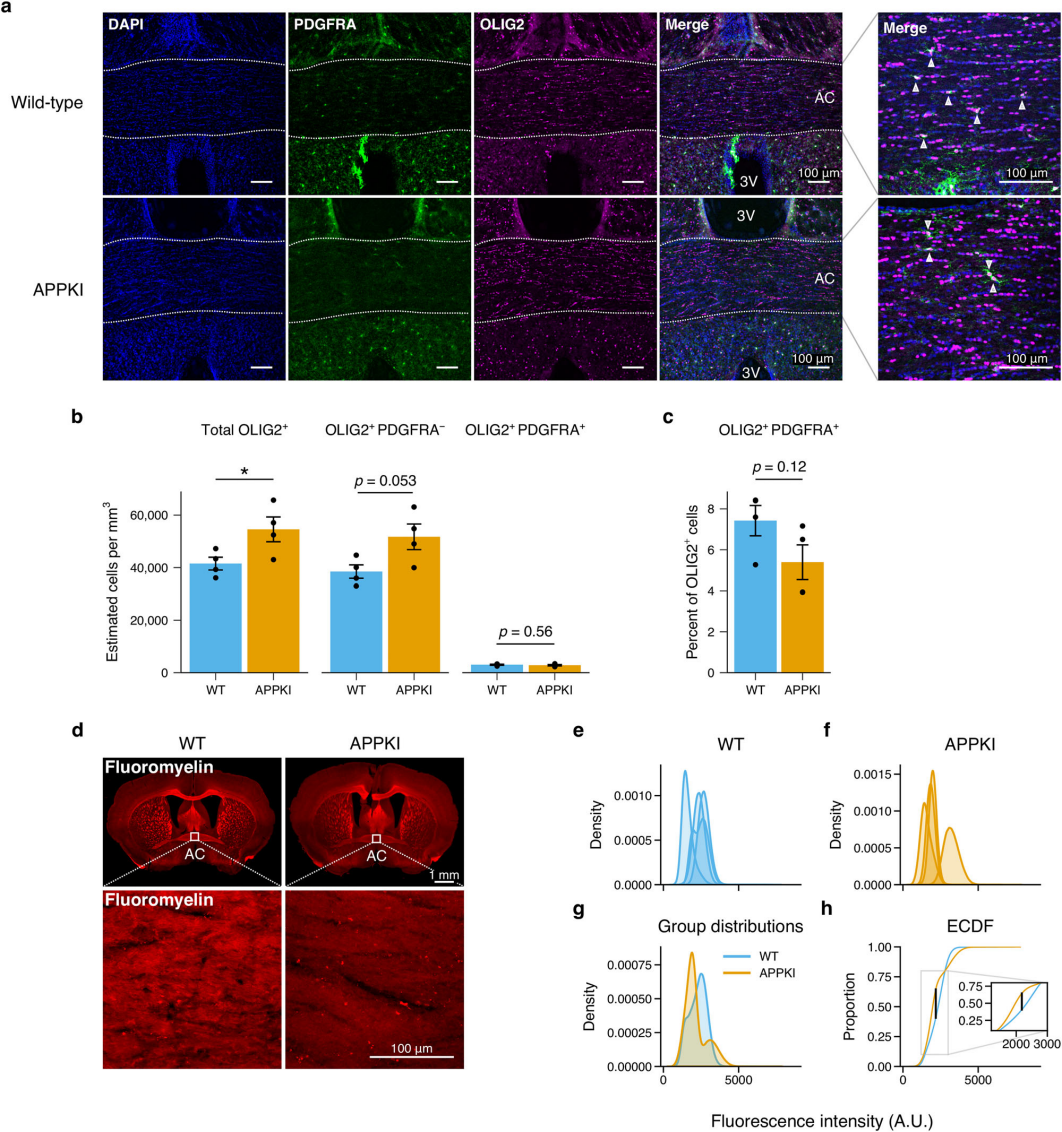
Oligodendrocyte and myelin quantification in the AC in middle age mice. ***a***, Representative confocal images of the midline AC for wild-type and APPKI mice. The AC is outlined in white. High magnification images are shown on the right. White triangles indicate representative OLIG2^+^ PDGFRA^+^ cells. AC: anterior commissure; 3V: third ventricle. Scale bars indicate 100 μm. ***b***, Stereological quantification of oligodendrocytes and OPCs. ***c***, Quantification of the percentage of OPCs of the total OLIG2^+^ population. ***b*** and ***c***, Points represent individual mice (*n* = 4 mice per group); bars represent mean; error bars represent SEM; blue: WT; orange: APPKI. Student’s independent *t*-test. *p < 0.05. Stereological quantification parameters are described in Extended Data Table 2-1. Individual measurements are described in Extended Data Tables 2-2 and 2-3. ***d***, Representative images of wild-type and APPKI mice stained with Fluoromyelin Red. White squares indicate the location of high magnification images used for quantification. AC: anterior commissure. Scale bars in top row indicate 1 mm. Scale bars in bottom row indicate 100 μm. ***e*** and ***h***, Quantification of Fluoromyelin Red. Kernel density estimation (KDE) of pixel intensity for each subject for wild-type (***e***) and APPKI (***f***) mice. ***g***, Pooled KDE for wild-type and APPKI mice. ***h***, Empirical cumulative distribution function (ECDF) for the pooled pixel values for each group. Black vertical line indicates KS test statistic. Inset depicts a zoomed view of the ECDF at the area of largest difference. A.U.: arbitrary units.

Since FA is traditionally used as a proxy of WM, we investigated whether there were changes in oligodendrocytes—the myelinating cells of the central nervous system—in APPKI mice in middle age, prior to the reduced FA observed in the DTI. In particular, we examined two of the regions observed to have reduced FA in APPKI mice (Fig. 1*g*–*h*): the AC, which is a prominent interhemispheric WM tract, and the hippocampus, because of its known vulnerability in AD. We performed blinded unbiased stereology to quantify the number of oligodendrocytes, characterized by the expression of the oligodendrocyte transcription factor 2 (OLIG2), and oligodendrocyte progenitor cells (OPCs), which co-express OLIG2 and the PDGFRA (Pringle and Richardson, 1993; Kuhn et al., 2019). We observed a statistically significant greater number of oligodendrocytes (Student’s independent *t*-test, *t*(6) = −2.454, *p* = 0.0495) and no statistically significant difference in the percentage of OPCs (Student’s independent *t*-test, *t*(6) = 1.81, *p* = 0.12; Fig. 2*a*–*c*, Extended Data Tables 2-2 and 2-3).

10.1523/ENEURO.0365-23.2023.t2-1 Table 2-1 Anterior commissure stereological quantification parameters. Download Table 2-1, XLSX file.

10.1523/ENEURO.0365-23.2023.t2-2 Table 2-2 Anterior commissure count estimates. Count: raw counts from serial sections. Estimated number of cells *N* calculated using Equation 1. CE: Gundersen’s coefficient of error (*m* *=* 1; Equation 2). Download Table 2-2, XLSX file.

10.1523/ENEURO.0365-23.2023.t2-3 Table 2-3 Anterior commissure estimated number of cells per mm^3^. Download Table 2-3, XLSX file.

After observing a higher number of oligodendrocytes in the AC in middle age APPKI mice, we asked whether APPKI mice also had a higher amount of myelin in the AC. To test this, we used the lipophilic dye Fluoroymelin Red and quantified the fluorescence intensity of the AC in both groups (Fig. 2*d*). Interestingly, we observed a reduction in Fluoromyelin Red intensity in APPKI mice (KS test, KS statistic = 0.238, *p* < 0.001; Fig. 2*e*–*h*). Together, these data suggest that, despite more oligodendrocytes, APPKI mice appeared to have less lipid content. This suggests that in middle age there may be an impairment in myelin homeostasis mechanisms in APPKI mice that deteriorates with age, leading to reduced FA observed in the late age DTI.

### APPKI mice have greater number of oligodendrocytes and higher oligodendrocyte-related protein expression in the hippocampus in middle age preceding observable DTI changes

**Figure 3.**
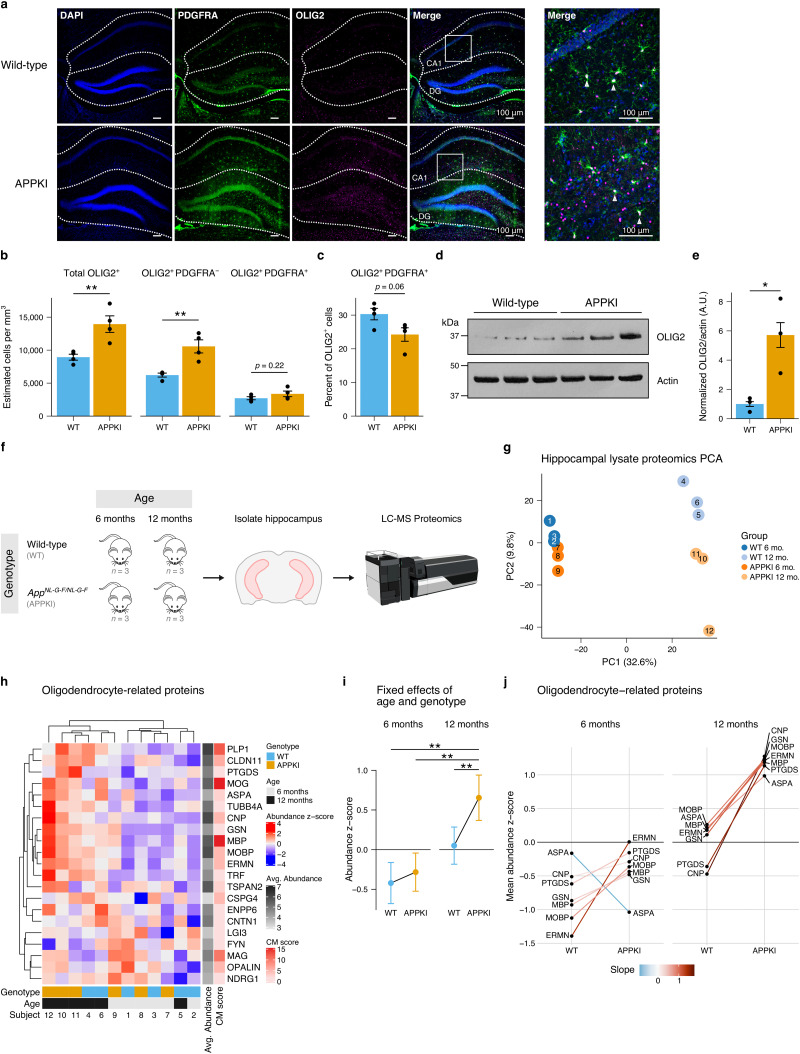
Oligodendrocyte quantification and proteomics in the hippocampus in middle age mice. ***a***, Representative confocal images of the hippocampus for 10-month-old wild-type and APPKI mice. The DG and CA1 of the hippocampus are outlined. The white squares indicate the location for the high magnification images on the right. White triangles indicate representative OLIG2^+^ PDGFRA^+^ cells. Scale bars indicate 100 μm. ***b***, Stereological quantification of oligodendrocytes and OPCs. ***c***, Quantification of the percentage of OPCs of the total OLIG2^+^ population. Stereological quantification parameters are described in Extended Data Table 3-1. Individual measurements are described in Extended Data Table 3-3. ***d***, Representative Western blot of hippocampal lysate probed for OLIG2 and actin. ***e***, Quantification of membrane in (***d***). A.U.: arbitrary units. ***b***, ***c***, and ***e***, Points represent individual mice (*n* = 4 mice per group). Bars represent mean. Error bars represent SEM. Student’s independent *t*-test. ^**^p < 0.01; *p < 0.05. ***f***, Experiment design for proteomics. Hippocampi were isolated from 6- and 12-month-old WT and APPKI mice and processed for LC-MS proteomics. Details regarding samples and abundnace measurements are described in Extended Data Tables 3-2, 3-4, and 3-5. ***g***, PCA of hippocampal lysate proteomics data obtained from 6- and 12-month-old WT and APPKI mice. Points represent individual subjects; numbers indicate subject ID. Individual PC percentages are shown in Extended Data Table 3-6. ***h***, Heatmap of oligodendrocyte-related proteins detected using the CM 2.0 database query for “mouse” + “brain” + “oligodendrocyte”. Color indicates the (row) standardized abundance for each protein for each subject. Gray row annotations indicate the average protein abundance across all subjects. Red row annotations indicate the CM score obtained from the CM database. Higher values indicate proteins most associated with the CM database query. ***i***, Fixed effects of age and genotype for LMM analysis of oligodendrocyte-related proteins. Points represent model fit. Error bars represent 95% CI. Significance code: **p < 0.01. ***j***, Slopegraph of oligodendrocyte-related proteins detected in proteomics dataset for 6-month-old and 12-month-old WT and APPKI mice with at least one *q* < 0.2 pairwise comparison. Points indicate mean abundance *z*-score for given protein for each group. Lines connect the same protein between WT and APPKI. Color of line indicates slope difference between WT and APPKI within each age group. Additional statistics are provided in Extended Data Table 3-(7-10).

Having observed a greater number of oligodendrocytes in the AC, we next investigated if there were alterations in oligodendrocytes in the hippocampus, which we also observed to have reduced FA in later age. We similarly performed unbiased stereology to quantify the number of oligodendrocytes and OPCs in the hippocampus. As in the AC, we observed a statistically significant greater number of total oligodendrocytes (Student’s independent *t*-test, *t*(6) = −3.75, *p* = 0.009) and putative mature oligodendrocytes (OLIG2^+^ PDGFRA^−^ cells; Student’s independent *t*-test, *t*(6) = −4.19, *p* = 0.006) in the hippocampus in 10-month-old APPKI mice compared to age-matched wild-type mice, and we observed a slight (not statistically significant) reduction in the percentage of OPCs (Student’s independent *t*-test, *t*(6) = 2.3068, *p* = 0.06; Fig. 3*a*–*c*, Extended Data Table 3-3). We further validated this observation at the protein level using Western analysis and observed a higher expression of OLIG2 in the hippocampus (Student’s independent *t*-test, *t*(6) = −3.15, *p* = 0.035; Fig. 3*d* and *e*).

10.1523/ENEURO.0365-23.2023.t3-1 Table 3-1 Hippocampus stereological quantification parameters. Download Table 3-1, XLSX file.

10.1523/ENEURO.0365-23.2023.t3-2 Table 3-2 TMT10plex™-labeling of peptides for 6- and 12-month-old hippocampal lysate samples. (Note that only six of the nine experimental samples in each kit were used in this study; the other three were used for another experiment.) Download Table 3-2, XLSX file.

10.1523/ENEURO.0365-23.2023.t3-3 Table 3-3 Hippocampus counts and estimated cells per mm^3^. Count: raw counts from serial sections. Estimated number of cells N calculated using Equation 1. CE: Gundersen’s coefficient of error (*m* = 1; Equation 2). Download Table 3-3, XLSX file.

10.1523/ENEURO.0365-23.2023.t3-4 Table 3-4 Abundance values from LC-MS proteomics experiment. Download Table 3-4, XLSX file.

10.1523/ENEURO.0365-23.2023.t3-5 Table 3-5 Pairwise group comparisons of protein abundnace levels from LC-MS proteomics experiment. Download Table 3-5, XLSX file.

10.1523/ENEURO.0365-23.2023.t3-6 Table 3-6 Variance explained by each principal component (PC) from hippocam­pus lysate proteomics data. Download Table 3-6, XLSX file.

10.1523/ENEURO.0365-23.2023.t3-7 Table 3-7 Fixed effects from linear mixed model results for oligodendrocyte-related proteins. Significance code: **p *<* 0.01; *p *<* 0.05. Download Table 3-7, XLSX file.

10.1523/ENEURO.0365-23.2023.t3-8 Table 3-8 Random effects from linear mixed model results for oligodendrocyte- related proteins. Download Table 3-8, XLSX file.

10.1523/ENEURO.0365-23.2023.t3-9 Table 3-9 *Post hoc* pairwise comparisons for oligodendrocyte-related protein linear mixed model. SE: standard error. Download Table 3-9, XLSX file.

10.1523/ENEURO.0365-23.2023.t3-10 Table 3-10 Pairwise comparison of proteins from proteomics data. Student’s inde­pendent f-test. p values were adjusted for multiple comparisons within each group using Benjamini- Hochberg’s false discovery rate (FDR). Included are proteins with *q* *<* 0.2. FC: fold change. SE: standard error. Download Table 3-10, XLSX file.

After observing both a greater number of oligodendrocytes and OLIG2 at the protein level in middle age APPKI mice (Fig. 3*b*–*e*), we explored protein expression near this middle age time window by performing LC-MS proteomics analysis of hippocampal lysate from 6- and 12-month-old WT and APPKI mice (Fig. 3*f*, Extended Data Tables 3-4 and 3-5). First, we computed PCA across all proteins detected for each of the four groups (Fig. 3*f*). The first principal component (PC1) accounted for over 30% of the variance in the data (Extended Data Table 3-6), and largely segregated the four groups according to age (Fig. 3*f*). The second principal component (PC2) accounted for 9.8% of the data (Extended Data Table 3-6) and segregated the four groups by genotype (Fig. 3*f*). This suggests that the largest differences in protein expression were contributed by age and genotype.

We sought to test the hypothesis that oligodendrocyte-related proteins are upregulated in APPKI mice. To do this, we used the CM 2.0 database (Hu et al., 2023) to query proteins associated with oligodendrocytes in the mouse brain. After cross-referencing this list with proteins detected in our sample, we compared the abundance values of proteins with the highest CM scores (i.e., proteins most associated with mouse oligodendrocytes, Fig. 3*h*). We used a LMM to test if there was a greater abundance of oligodendrocyte-related proteins in APPKI mice. There was a statistically significant main effect of age (*t*(22.56) = 2.408, *p* = 0.0037) and a statistically significant interaction between age and genotype (*t*(91.53) = 2.31, *p* = 0.0231; Extended Data Tables 3-7 and 3-8; Fig. 3*i*). *Post hoc* pairwise comparisons showed a statistically significant difference in oligodendrocyte-related protein abundance between 6-month-old WT and 6-month-old APPKI mice (*t*(20) = −4.506, *p* = 0.0011), 12-month-old WT and 12-month-old APPKI mice (*t*(20) = −3.606, *p* = 0.0088), and between 6-month-old APPKI and 12-month-old APPKI mice (*t*(20) = −4.307, *p* = 0.0018; Extended Data Table 3-9). At the level of individual proteins, using a threshold of *q* < 0.2, there was a statistically significant difference in protein abundance in Ermin (ERMN; 6-month-old: APPKI/WT; 12-month-old: APPKI/WT), $2^{\prime },3^{\prime }$-cyclic-nucleotide 3^′^-phosphodiesterase (CNP), gelsolin (GSN), myelin-associated oligodendrocyte basic protein (MOBP), prostaglandin D_2_ synthase (PTGDS; 12-month-old: APPKI/WT), aspartoacylase (ASPA), MBP (APPKI: 12-month-old/6-month-old; statistical details provided in Extended Data Table 3-10). Together, this suggests that the number of oligodendrocytes and multiple oligodendrocyte-related proteins are higher in the hippocampus in middle age APPKI mice prior to structural deficits observed at the macroscale by DTI.

## Discussion

In this study, we used the APPKI mouse model (Saito et al., 2014) that harbors three FAD mutations to study structural alterations of the brain across age. We observed reduced FA overall in APPKI and late age mice, especially in the AC, hippocampus, hypothalamus, and neocortex, suggesting that there were microstructural impairments in those regions. Furthermore, Fluoromyelin quantification in the AC suggested that there was reduced myelin content in the APPKI compared to wild-type. To investigate this further at the cellular level, we observed that, by middle age, APPKI mice have a greater oligodendrocyte population in both the hippocampus and the AC. There was also a slight, but not statistically significant (*p* = 0.06), reduction in the percentage of OPCs in the hippocampus. At the molecular level, proteomics analysis of hippocampi from WT and APPKI mice showed a higher expression of oligodendrocyte-related proteins in 12-month-old mice compared to 6-month-old mice, and a significant interaction between age and genotype. Together, this implies that APPKI mice show WM-related alterations by middle age, prior to observed changes by DTI, and that the higher production of oligodendrocytes and oligodendrocyte-related proteins suggests an impaired homeostasis or regulation of oligodendrocytes and myelin.

Previous studies have examined different rodent models of AD using MRI and DTI. Groups that have studied rodents at different age points have generally observed diffusion measurements consistent with measures of microstructural impairment in human AD patients, such as reduced FA, increased radial diffusivity or mean diffusivity, atrophy in GM, and enlargement of the lateral ventricles (Nir et al., 2013; Weston et al., 2015). In GM, changes in volume or DTI measures in the mouse cortex and hippocampus have been reported (Sun et al., 2005; Lau et al., 2008; Qin et al., 2013; Badea et al., 2016; Anckaerts et al., 2019). In WM, changes have been observed in the AC (Sun et al., 2005; Lau et al., 2008; Sahara et al., 2014), internal capsule (Lau et al., 2008; Qin et al., 2013; Badea et al., 2016), external capsule (Anckaerts et al., 2019; Nie et al., 2019), and corpus callosum (Sun et al., 2005; Lau et al., 2008; Anckaerts et al., 2019). The changes observed in DTI begin to appear around middle age, between approximately 8 and 12 months, which is similar to our results where changes in FA were observed after 10 months. While FA is widely used, other measures of tissue integrity including myelin water fraction (MacKay and Laule, 2016), magnetization transfer ratio (Moccia et al., 2020), and neurite orientation dispersion and density imaging (Zhang et al., 2012) may also be informative. Additionally, while our results were obtained *in vivo*, *ex vivo* MRI studies have the potential to offer much higher resolution that can only be achieved with longer acquisition times. Future work combining *ex vivo* imaging and structural connectomics may offer more insight into the network connectivity of the brain in addition to traditional MRI tissue and diffusion measures.

Aging is known to affect both myelin and oligodendrocytes in many brain regions. Across childhood development, Baum et al. (2022) observed that an MRI proxy of myelination was found to increase in a region-specific manner based on their sensorimotor—association axis ranking, such that sensorimotor regions myelinate earlier and association regions myelinate later in adolescence. In the adult, myelination continues and remains relatively stable until approximately 40–50 years of age, after which there is a notable decline, the rate of which is also region-specific (Bartzokis, 2004, 2011; Langnes et al., 2020). In AD, the pattern of degeneration is notably inverse of the developmental myelination process (Braak et al., 2006), suggesting that association areas that myelinate later in life are the earliest areas of vulnerability in AD (Braak et al., 2006; Bartzokis, 2011).

The microscopic changes in WM likely occur many years before gross changes detected by MRI and the onset of cognitive impairment (Bartzokis, 2004; Jack et al., 2013). Sandell and Peters (2003) observed that the area of the AC in rhesus monkeys, for example, peaks in middle age, but by old age it decreases to below that of young monkeys. At the cellular level, aging leads to an increase in both the density and number of oligodendrocytes in multiple areas of the brain (O’Kusky and Colonnier, 1982; Peters and Sethares, 2004; Peters et al., 2008). Despite the increase in the number of oligodendrocytes, electron microscopy of the AC showed a decrease in the number of myelinated fibers, increase in the number of degenerated axons, and increase in the number of myelin structural alterations with increasing age. Similarly, we did not observe any gross changes in FA until after middle age. This is consistent with both histological and electron microscopy studies in rhesus monkeys as well as the human neuroimaging studies mentioned earlier. In addition to structural changes, there is evidence to suggest that the number of myelinated axon fibers positively correlates with cognitive performance (Sandell and Peters, 2003). Together, these data suggest that alterations in myelin quality, structure, and oligodendrocyte dynamics likely begin in middle age. In the context of AD, the number of oligodendrocytes in APP/PS1 mice were found to be comparable at 3 months of age in GM but significantly increased in GM by 6 months of age (Behrendt et al., 2013). A study by Chen et al. (2021) observed a comparable number of NG2^+^ OLIG2^+^ OPCs at 8 months of age in APP/PS1 mice. They also observed more newly formed myelin and degenerated older myelin in APP/PS1 mice compared to controls. Our results with the APPKI are largely consistent with these results; namely, we observed a greater number of oligodendrocytes in middle age APPKI mice and reduced lipid content, which could be indicative of reactive oligodendrocytosis in response to injury (Levine et al., 2001). One interpretation of this could be an increased attempt at myelin repair (Bartzokis, 2011; El Waly et al., 2014), which may be impaired based on the observed reduction of Fluoroymelin fluorescence intensity, in combination with increasing amyloid plaque deposition (Behrendt et al., 2013; Chen et al., 2020), especially given the increased vulnerability of late-myelinating regions (Sachdev et al., 2013; Baum et al., 2022).

Furthermore, the expression of multiple oligodendrocyte-related proteins appeared to be altered in middle age APPKI mice, in particular, ERMN, CNP, GSN, MOBP, PTGDS, ASPA, and MBP. ERMN is a cytoskeletal protein expressed exclusively in CNS myelin, and is thought to aid the arrangement, compaction, and maintenance of myelin in oligodendrocytes (Brockschnieder et al., 2006), and was found to be greater in APPKI relative to WT for both the 6- and 12-month-old groups. CNP is an intracellular protein expressed in oligodendrocytes. It is known to regulate oligodendrocyte differentiation and plays a role in the extension of oligodendrocyte processes for myelin development (Gravel et al., 1996). In the CNS, GSN is expressed predominantly by oligodendrocytes, and is involved in severing and restructuring actin filaments (Tanaka and Sobue, 1994; Vouyiouklis and Brophy, 2002). It is thought to have a role in the formation of myelin (Vouyiouklis and Brophy, 2002). Notably, extracellular GSN has also been shown to interact with amyloid β (Chauhan et al., 1999). MOBP is involved in myelin compaction, stability, regulation of processes, and oligodendrocyte morphology (Gould et al., 2002; Montague et al., 2006; Schäfer et al., 2016). PTGDS is responsible for the production of prostaglandin D_2_ in the brain, and has also been shown to be expressed in OPCs (Sakry et al., 2015). ASPA is an enzyme that catalyzes N-acetylaspartic acid into aspartate and acetate, which is critical for the biosynthesis of myelin lipids, and thus is important for WM integrity (Chakraborty et al., 2001; Bitto et al., 2007; Wijayasinghe et al., 2014). Taken together, the alterations in these oligodendrocyte-related proteins suggest that multiple characteristics including oligodendrocyte morphology, processes, and myelin maintenance are affected in later age and APPKI. Future studies using both male and female mice will be informative to know if there are sex-specific differences in the expression of these proteins, since there are known sex differences in oligodendrocyte dynamics (Cerghet et al., 2006) and AD (Li and Singh, 2014; Mazure and Swendsen, 2016). Additionally, future studies could also address the functional implications of these changes in oligodendrocytes and the possible mechanism by which this occurs, as well as in other areas of the brain.

In summary, we used a combination of DTI, molecular biology, and proteomics to investigate temporal changes in WM using the *App^NL-G-F/NL-G-F^* mouse model of AD. We observed alterations in oligodendrocyte homeostasis and expression profile in the hippocampus in middle age, preceding reduced integrity measured by DTI in later age. Together, these results provide potential therapeutic biomarkers and a better understanding of the early progression of AD.

## Synthesis

Reviewing Editor: Selva Baltan, Oregon Health & Science University Department of Surgery Decisions are customarily a result of the Reviewing Editor and the peer reviewers coming together and discussing their recommendations until a consensus is reached. When revisions are invited, a fact-based synthesis statement explaining their decision and outlining what is needed to prepare a revision will be listed below. The following reviewer(s) agreed to reveal their identity: NONE. Note: If this manuscript was transferred from JNeurosci and a decision was made to accept the manuscript without peer review, a brief statement to this effect will instead be what is listed below.

Dear Authors,

Your manuscript has been evaluated as a "significant improvement to the current knowledge on AD". Several minor revisions are requested. The revised version will be reviewed by the Handling Editor only. A quick turnaround will be greatly encouraged.

Thanks.
